# Par-3 partitioning defective 3 homolog *(C. elegans) *and androgen-induced prostate proliferative shutoff associated protein genes are mutationally inactivated in prostate cancer cells

**DOI:** 10.1186/1471-2407-9-318

**Published:** 2009-09-08

**Authors:** Dimiter Kunnev, Igor Ivanov, Yurij Ionov

**Affiliations:** 1 Department of Cancer Genetics, Roswell Park Cancer Institute, Buffalo, New York, 14263, USA

## Abstract

**Background:**

Gene identification by nonsense-mediated mRNA decay inhibition (GINI) has proven its usefulness in identifying mutant genes in cancer cell lines. An increase in transcription in response to NMD inhibition of a subset of genes is a major cause of false positives when genes are selected for sequencing analysis. To distinguish between mRNA accumulations caused by stress response-induced transcription and nonsense-containing mRNA stabilizations is a challenge in identifying mutant genes using GINI.

**Methods:**

To identify potential tumor-suppressor genes mutated in prostate cancer cell lines, we applied a version of GINI that involves inhibition of NMD in two steps. In the first step, NMD is inhibited in duplicate tissue-culture plates. During this step, both the substrate for NMD and stress-response mRNA transcripts are accumulated in cells. In the second step, transcription is inhibited in both plates and NMD is inhibited in one plate and released in the second plate. Microarray analysis of gene-expression profiles in both plates after the second step detects only the differences in mRNA degradation but not in mRNA accumulation.

**Results:**

Analyzing gene expression profile alterations in 22RV1 and LNCaP prostate cancer cells following NMD inhibition we selected candidates for sequencing analysis in both cell lines. Sequencing identified inactivating mutations in both alleles of the PARD3 and AS3 genes in the LNCaP and 22RV1 cells, respectively. Introduction of a wild-type PARD3 cDNA into the LNCaP cells resulted in a higher proliferation rate in tissue culture, a higher adhesion of LNCaP cells to the components of extracellular matrix and impaired the growth of the LNCaP cells in soft agar and in a three-dimensional cell-culture.

**Conclusion:**

The mutational inactivation in a prostate cancer cell line of the PARD3 gene involved in asymmetric cell division and maintenance of cell-polarity suggests that the loss of cell-polarity contributes to prostate carcinogenesis.

## Background

Inactivation of tumor-suppressor genes in cancer cells frequently occurs through the nonsense mutation in one allele and the loss of the chromosome locus containing the wild type allele. Identifying the nonsense mutations in the remaining allele in regions of frequent losses of heterozygosity in tumors indicates putative tumor suppressor genes. Nonsense mutations located in mRNA sequences more than 22 nucleotides upstream of the last exon/exon junction elicit a rapid degradation of mutant mRNA through the nonsense-mediated mRNA decay (NMD) pathway [1,2]. Since triggering the NMD of mutant mRNA requires an initial round of translation, blocking translation with specific drugs, such as emetine, has been shown to abrogate the NMD-mediated degradation of mutant mRNAs [3]. This results in an increased amount of mRNA transcripts from genes containing nonsense or frameshift mutations, which can be detected using gene-expression microarrays. A strategy has been proposed for the identification of genes containing nonsense or frameshift mutations [4] using microarray analysis of mRNA profile alterations resulting from inhibiting NMD in cell lines (GINI).

The major complication in identifying mutant genes using GINI is the fact that too many genes that do not contain nonsense mutations show mRNA accumulation following the blocking of NMD with emetine or small interfering RNA against hUpf1 and hUpf2 genes, the major regulators of NMD. Part of these false-positive candidates is represented by genes transcriptionally induced by stress response to inhibition of NMD and the other part is represented by a natural substrate for NMD genes. These are the genes that have an upstream open reading frame in their 5' untranslated region, introns in the 3' untranslated region and the products of alternative splicing that produce nonsense codons or frameshifts [5]. Using control cell lines helps to eliminate false-positives represented by the natural substrate of NMD genes but, due to the variability in stress response between different cancer cell lines, does not efficiently eliminate false-positive candidates produced by stress response. Combining GINI microarray analysis with array-based comparative genomic hybridization (aCGH) has been proposed for the genome-wide identification of genes with biallelic inactivation involving nonsense mutations and loss of the wild-type allele. Although this approach led to identification of a previously unknown mutation in the receptor tyrosine kinase gene EPHB2 in the DU145 prostate cancer cell line [6], combining GINI and aCGH is not the best strategy for prostate cancer cells. The majority of prostate cancer cell lines are known to have microsatellite instability (MSI), which is caused by the inactivation of components of DNA mismatch repair (MMR) in prostate cells [7]. Inactivation of MMR causes a high rate of replication errors resulting in an elevated frequency of mutations. An inverse correlation between MSI and LOH reported for colorectal cancer [8] suggests that in cancers with MSI the inactivation of a tumor-suppressor gene is more likely to occur by two independent mutations in two alleles rather than by mutation in one allele and the loss of the other. Of five cell lines the most frequently used for prostate cancer research experiments PC-3, LNCaP, DU-145, LAPC-4, and 22RV1 only PC-3 does not have microsatellite instability. Therefore, relying upon aCGH analysis when choosing candidates for sequencing out of list of genes generated by GINI analysis of MSI positive prostate cancer cell lines will result in missing genes inactivated by two independent mutations.

In our lab, we have developed a modification of GINI that allows us to eliminate from further analysis the candidate genes that show mRNA increases due to stress response-induced transcription [9]. The idea behind the modification is use microarray analysis to measure only mRNA degradation rather than the sum of two processes, mRNA degradation and mRNA accumulation, caused either by mutant mRNA stabilization or by the increased transcription of stress-induced genes. In this work, we applied our method to LNCaP and 22RV1 prostate cancer cell lines with MSI, and identified previously unknown biallelic inactivating mutations in the par-3 partitioning defective 3 homolog (PARD3) gene in LNCaP cells and the androgen-induced prostate proliferative shutoff associated protein (AS3) gene in 22Rv1 cells.

## Methods

### Cell culture

The LNCaP and 22Rv1 prostate carcinoma cell lines (ATCC) were grown in RPMI-1640 supplemented with 10% fetal bovine serum (FBS), 100 U/ml penicillin and 0.1 mg/ml.

### GINI analysis

Cells were seeded in four tissue-culture plates. Caffeine (10 mM) was added to three plates and one plate was incubated without caffeine as a control. Following four hours' incubation, the medium was removed from one plate (with caffeine) and from the control plate and total RNA was prepared using TRIZOL reagent, according to manufacturer's instructions, and used for an Affymetrix U133Plus2.0 oligonucleotide array hybridization. The caffeine-containing medium of the two remaining plates was removed, the cells were washed twice with phosphate-buffered saline and the medium with actinomycin D (2 *μ*g/ml) together with caffeine (10 mM) was added to one plate. Actinomycin D alone was added to the other plate. Following another four hours' incubation, the total RNA from both plates was prepared and used for an Affymetrix U133Plus2.0 oligonucleotide array analysis.

### Affymetrix oligonucleotide array data analysis

Messenger RNA levels in drug-treated and untreated cells were measured using the Affymetrix U133Plus2.0 array. The results of array hybridization presented as CEL files were imported into the Bioconductor system using the R software package. Convolution background correction, quantile normalization and a summarization of probe intensity were processed using an adaptation of the Robust Multi-Chip Average (gcRMA) [10]. To compute GCRMA expression values, we conducted a *gcrma *function of the Bioconductor package, publicly available at the http://www.bioconductor.org. The quantile normalized log transformed GCRMA values were than used to select candidate targets for sequencing analysis using selection criteria described in the "Result" section of the text. The Affymetrix gene expression data were submitted to Gene Expression Omnibus (GEO) database under accession number GSE16856.

### Array comparative genomic hybridization

Genomic pooled normal control DNA and DNA from LNCaP or from 22Rv1 cells were fluorescently labeled by random priming and were hybridized to RPCI arrays containing ~6,000 RPCI-11 BAC clones, as described [11]. The hybridized slides were scanned using a Genepix 4200A scanner (Axon, Inc., Union City, CA) to generate high-resolution (10 μm) images for both Cy3 and Cy5 channels, and image analysis was performed using ImaGene (version 4.1) software (BioDiscovery, Inc., El Segundo, CA).

### Sequencing analysis

One microgram of total RNA from caffeine-treated prostate cancer cells was reverse transcribed using the SuperScript II protocol (Invitrogen, Carlsbad, CA, USA). Overlapping PCR primer sets were used to generate products spanning the entire open reading frames for candidate genes. Primers for sequencing analysis were designed using Primer3 software available online http://frodo.wi.mit.edu/primer3/. The PCR products were gel-purified and sequenced using the Applied Biosystems' PRISM 3100 Genetic Analyzer.

### Cloning and expression of PARD3

Reverse transcribed RNA from MCF-10A cells (ATCC) was used to amplify PARD3 cDNA by PCR with AccuPrime Pfx DNA Polymerase (Invitrogen) with the following primer sequences: Forward-gccaccATGAAAGTGACCGTGTGCTTCG; Reverse-TCACTTATCGTCGTCATCCTTGTAATCTCTCTTCTCGGGCTTCAGTTTGGC. The primers were designed with additional flanking sequences for Kozak (for the forward primer) and the FLAG-tag sequence followed by stop codon (for the reverse primer). The product was cloned in a pCR-Blunt II TOPO (Invitrogen) vector by using the Zero Blunt TOPO PCR Cloning Kit (Invitrogen). The sequence and direction of the insert was verified by sequencing. The PARD3 cDNA with Kozak and Flag-tag sequences attached was sub-cloned into a pBMN-I-GFP retroviral vector (a gift from Dr. G.Nolan from Stanford University), at the EcoRI site.

The pBMN-I-GFP-PARD3-FLAG construct was used for retroviral production according to the protocol described in the manual available at http://www.addgene.org/Garry_Nolan, based on the packaging cell line Phoenix (authorized for Roswell Park Cancer Institute). The same procedure was applied to the pBMN-I-GFP control vector without an insert. The virus containing medium was collected, filtrated with 0.4 μm Super Membrane Syringe Filter (Pall Corporation), concentrated by VIVASPIN 20 with membrane 50,000 MWCO PES (sartorius sedim biotech) and used for infection of the LNCaP cells. A week later the cell was subjected to GFP cell-sorting with FACS. The Par3 protein expression was confirmed by immunoblotting with an anti-FLAG mouse monoclonal antibody (Sigma) as well as with anti-Par3 mouse polyclonal antibody (Abcam).

### Fluorescence microscopy

Cells were grown in a medium containing 10% FBS on glass coverslips (22 mm × 22 mm). The cells was washed with PBS and fixed with 4% paraformaldehyde, permeabilized with 0.05% Triton X-100 for 10 min and blocked with 3% milk in PBS for 30 min. The anti-Flag antibody was added for one hour at a dilution of 1:300. A second antibody, goat anti-mouse Alexa Fluor 568 (Texas Red), was incubated for one hour at a dilution of 1:500. All incubations were done at room temperature. Fluorescent images were captured using a Nikon TE2000-E inverted microscope equipped with Roper CoolSnap HQ CCD camera.

### Western blotting

The whole-cell lysates from cultured cells were prepared with RIPA lysis buffer (50 mM Tris.HCl, pH 8.0, 150 mM NaCl, 1% Igepal CA 630, 0.5% sodium deoxycholate, 0.1% SDS) supplemented with a protease inhibitor cocktail and phosphatase inhibitor cocktails I and II (Sigma). The samples were sheared by 5-10 passes through a 27 g needle incubated on ice for 40 min. Extracts were cleared by centrifugation (30 min, 10,000 g, 4°C). Protein concentrations were determined by Bicinchoninic acid (BCA) protein assay reagent (Pierce) and lysates were boiled in a Laemmli samples buffer for 5 min before being subjected to SDS-PAGE. Equal amounts of proteins were separated using 10% SDS-polyacrylamide mini-gels. The proteins were transferred to the nitrocellulose membrane and stained with 0.1% Fast Green (Sigma) to confirm equal loading and even transfer. Membranes were incubated overnight with primary antibodies against Flag-tag (F 1804, mouse monoclonal M2, 1:1000, Sigma), anti-Par3 (Abcam, ab21987) or actin (A2066, rabbit polyclonal, 1:5000, Sigma), followed by incubation with respective horseradish peroxidase-conjugated secondary antibodies (sheep anti-mouse, 1:2000, Amersham). Immune complexes were visualized using the Super Signal chemiluminescence system (Pierce) and CL-XPosure film (Pierce).

### Cell proliferation assay

5 × 10^3 ^cells per well were plated in a 24-well format tissue culture plate in RPMI 1640 containing 5% fetal calf serum (FBS). Cells were cultured for the indicated time periods, trypsinized and counted by haemocytometer. The cell number was shown as the average ± S.D. of counting four individual wells per time-point. The experiment was repeated independently three times with similar results.

### Cell adhesion assay

The 96-well plates were pre-coated with 10 μg/ml (100 μl) fibronectin (Sigma) or type I collagen (Calbiochem) in PBS. Wells were blocked with 10 mg/ml BSA in PBS and washed with PBS. Cells were seeded to the wells at 2 × 10^4^/well in a tissue-culture medium. The plate was incubated for three hours in a CO_2 _incubator at 37°C. Nonadherent and loosely attached cells were removed from the wells by gently washing them with DPBS (Cellgro) and the attached cells were fixed with 5% glutaraldehyde (E.M.S.). Attached cells were counted by microscope at 200× magnification.

### Colony formation in soft agar assay

To assess the anchorage-independent growth of cells in soft agar, 5 × 10^3 ^cells were suspended in 2 ml of 0.4% L.M.P. agarose (Ultra Pure, Invitrogen) (top agar) in complete growth media and plated in six-well plates pre-coated with 2 ml of 0.8% L.M.P. agarose (base agar) in the same medium. The cells were then overlaid with 2 ml of complete growth media, which was changed every six days. Three weeks after starting the culture, the colonies were stained with 0.005% crystal violet in 25% methanol and colonies were counted visually.

### Three-dimensional culture

Three-dimensional cultivation of LNCaP cells was performed, as described in [12]. To the wells of 24-well plates, 200 μl of phenol red-free ice cold Matrigel were added and the plates were placed in a 37°C incubator for one hour to allow the matrix to gel. LNCaP cells were seeded onto this bed as a single-cell suspension in a tissue-culture medium containing 2% Matrigel. After Matrigel polymerization at 37°C, the tissue-culture medium was overlaid over polymerized Matrigel and the medium was changed every 48 hours.

## Results

### Identifying mutant genes in prostate cancer cell lines using GINI analysis

In the original GINI protocol, the mRNA level alterations detected by microarray hybridization after inhibition of NMD in cells represent the result of two simultaneous processes, i.e., mRNA synthesis and mRNA degradation. The inability to distinguish between mRNA accumulation caused by an induced novel mRNA synthesis and that caused by blocking mRNA degradation is the major source of false-positives produced by GINI analysis. Our modified GINI protocol allows separation of these two processes and analysis only mRNA degradation following NMD inhibition. The protocol involves two consecutive steps of NMD inhibition. In the first step, NMD is inhibited in duplicate tissue-culture plates of each analyzed cell line. During this step, the accumulation of mRNA transcripts of stress-response genes due to enhanced transcription as well as of genes representing a natural substrate of NMD (including those containing nonsense mutations) takes place. During the second step, transcription is blocked with actinomycin D in both tissue-culture plates, and NMD inhibition is continued in one tissue-culture plate and released in the other for each analyzed cell line. During this step, the natural substrate of NMD, including nonsense mutation-containing transcripts, are degraded at different rates between the two plates, depending on the NMD status in each plate, while the stress response genes should be degraded with the same efficiency regardless of NMD status, Comparing mRNA levels after the second step of NMD inhibition using Affymetrix Genechip hybridization allows measurement of the degree of mRNA degradation when NMD is either "on" or "off", without the interference of stress response-induced mRNA synthesis. We applied this strategy to identify mutant genes in the MSI -positive 22Rv1 and LNCaP prostate cancer cell lines, also using these cell lines as controls against each other, assuming that different genes were mutated in these cell lines. To identify nonsense or frameshift mutation-containing genes in LNCaP and 22Rv1 cells, we performed the following microarray gene expression profiling experiments: Using Affymetrix Plus2.0 Genechip hybridization, we measured mRNA levels in LNCaP and 22Rv1 cells at the following times- 1) before inhibition of NMD, 2) after inhibiting NMD for four4 hours by incubating cells in a medium containing caffeine (We used caffeine instead of emetine for NMD inhibition because NMD is quickly restored in the cells after removal of caffeine but not of emetine from the media [9].), 3) after further inhibiting both NMD and transcription by incubating cells in a medium containing both caffeine and actinomycin D for four hours or after inhibiting transcription only by incubating cells in the medium with actinomycin D but without caffeine. Additional file 1: Table S1 shows the quintile normalized log2 transformed probe hybridization signal values of all performed microarray experiments.

To select candidates for sequencing out of a large list of LNCaP genes cells showing mRNA alterations following various modes of NMD inhibition, we applied the following arbitrarily chosen cut-off thresholds to the quintile normalized and log-transformed values of Affymetrix Genechip hybridization signal intensities. First, the mRNA level for a candidate gene in untreated cells before NMD inhibition was more than four-fold lower (< -2 on the log scale) in LNCaP cells than in 22Rv1 cells (assuming that NMD degrades mutant mRNA transcript in LNCaP cells). Second, the increases in mRNA levels following inhibition of NMD with caffeine in the first step were more than threefold (>1.3 on the log scale) in LNCaP cells and less than twofold (< 1.0 on the Log scale) in 22Rv1 cells. And, finally, the mRNA level for a candidate gene in LNCaP cells after the second step of NMD inhibition, i.e., after blocking both NMD and transcription, was more than threefold higher than after blocking transcription only, while in 22Rv1 cells the difference after the second step was chosen to be less than two fold. The candidates for the 22Rv1 cells were selected using the same cut-off thresholds as for the LNCaP cells.

**Figure 1:**
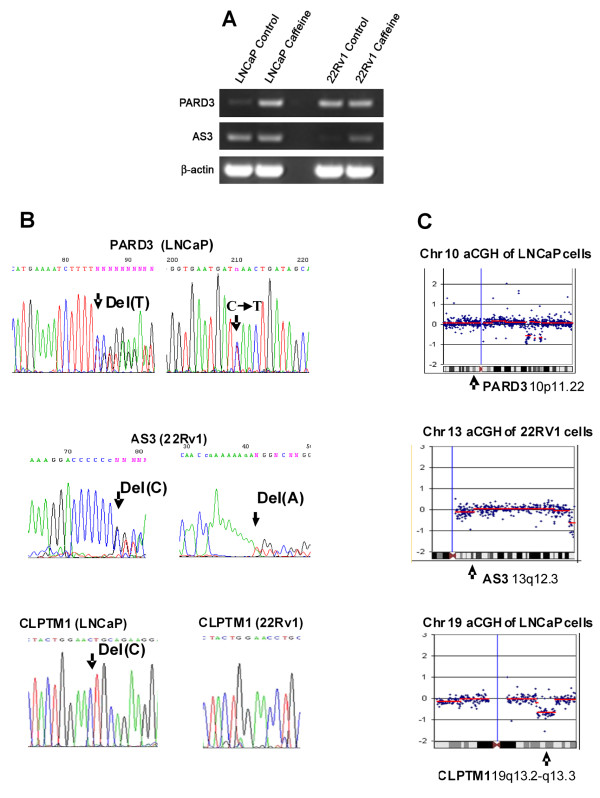
**Heterozygous bi-allelic inactivating mutations in PARD3 and AS3 genes in the LOH -free regions in LNCaP cells**. (A). RT-PCR analysis shows the lower mRNA levels in the untreated and the increased levels in caffeine treated LNCaP and 22Rv1 cells for the PARD3 and for AS3 genes, respectively. (B). Sequencing chromatograms show heterozygous deletion of a T in the (T)5 coding repeat in one allele and C to T substitution resulting in the TAA stop codon in the other allele of the PARD3 gene in LNCaP cells (top); heterozygous deletion of a C in the (C)7 repeat in one allele and of an A in the (A)9 repeat in the other allele of the AS3 gene in the 22Rv1 cells (middle); and a homozygous deletion of a C in the (C)2 repeat of the CLPTM1 gene in the LNCaP cells (bottom). (B). Array CGH analysis shows normal genomic content in the PARD3 and AS3 loci in the LNCaP and 22Rv1 cells and the loss of heterozygosity for the CLPTM1 locus in LNCaP cells.

Analyzing the quintile normalized, log-transformed Affymetrix hybridization data we identified 7 and 5 hybridization probes for the LNCap and 22Rv1 cells respectively that satisfied the chosen cut-off thresholds (Table 1). RT-PCR analysis (Figure 1A) shows that PARD3 and AS3 mRNA levels were lower in the LNCaP and in 22Rv1 cells respectively before NMD inhibition and were up-regulated in these cells following NMD inhibition with caffeine. By sequencing the cDNA corresponding to the identified probes, we found mutations in the PARD3 and AS3 genes in the LNCaP and 22Rv1 cells respectively. The homozygous frameshift mutation in another selected candidate in the LNCaP cells, in the cleft lip and palate-associated transmembrane protein 1 (CLPTM1), has been identified previously [13]. Figure 1 shows that inactivation of the PARD3 and AS3 genes in the LNCaP and 22Rv1 cells occurred through two independent mutations in both alleles of genes located in the regions of the cell genome identified by aCGH analysis as LOH-free (Figure 1C). The inactivation of the CLPTM1 gene in LNCaP cells on the other hand, most likely had occurred by mutation in one allele and the loss of the other as suggested by the gene's location in the region of LOH (Figure 1C). This result illustrates that combining GINI and aCGH helps to narrow down the number of candidates for sequencing but may result in missing genes inactivated by two independent mutations. It also shows the capability of the GINI to efficiently identify mutant genes regardless of the information on LOH.

**Table 1:** Candidates for sequencing analysis in the LNCaP and 22Rv1 selected using GINI.

	GENE Symbol	Probe set ID	22Rv1			LNCaP		
			Log_2 _(signal)	Log_2 _Accumulation	Log_2 _Degradation	Log_2 _(signal)	Log_2 _Accumulation	Log_2 _Degradation
	PARD3	221526_x_at	7.1	0.9	0.1	3.2	2.9	1.6
	ANKRD28	213035_at	8.2	0.4	0.4	5.6	1.7	1.5
	CLPTM1	211136_s_at	9.7	-0.5	-0.6	4.3	1.6	1.8
**LNCaP candidate**	VRK3	221999_at	6.5	0.3	0.5	3.9	1.5	2.5
**s**	GTPBP2	221050_s_at	8.2	0.2	0.2	5.2	1.5	2.1
	LOC692247	1561355_at	6.9	0.1	0.3	3.9	1.5	1.5
	AFF1	227198_at	10.5	-0.6	0.7	5.4	1.3	1.4
	AS3	207956_x_at	5.9	2.6	1.4	10.0	0.1	0.3
**22RV1**	TTLL7	219882_at	3.6	2.0	1.4	7.6	0.3	0.9
**candidate**	PDSB5	204742_s_at	3.9	1.9	1.3	7.8	-0.6	-0.6
**s**	EIF5	208708_x_at	9.2	1.7	2.5	11.3	0.7	0.9
	EIF2C2	225827_at	3.5	1.6	1.9	6.1	-0.6	0.1

RNAs from LNCaP and 22Rv1 cells were analyzed using Afffymetrix GeneChip Human Genome U133Plus 2.0 Array hybridization after the following treatments: 1) no treatment, 2) incubation with caffeine for four hours, 3) incubation with caffeine and actinomicin D following caffeine treatment for four hours and 4) incubation with actinomycin D following caffeine treatment for four hours. The candidates for sequencing analysis for LNCaP cells were selected by sorting the normalized, Log2 transformed hybridization signal values data using the following criteria: 1) expression of the candidate gene in the untreated LNCaP cells is more than four-times lower than in the untreated 22Rv1 cells used as a control, i.e., Log2(signal LNCaP) - Log2(signal 22Rv1) <2.0; 2). The mRNA level accumulation for candidate genes following incubation with caffeine for four hours should be more than threefold and less than twofold in the LNCaP and 22Rv1 cells, respectively: i.e., Log2 (Accumulation LNCaP) >1.3 and Log2(Accumulation 22Rv1) <1.0; 3). The mRNA levels for candidates after inhibiting both mRNA synthesis and mRNA degradation with actinomycin D and caffeine should be more than threefold and less than twofold higher than after inhibiting mRNA synthesis only with actinomycin D for the LNCaP and 22Rv1 cells, respectively: i.e., Log2 (Degradation LNCaP) >1.3 and Log2 (Degradation22Rv1) <1.0. Candidates for the 22Rv1 cells were selected in the same way, using the LNCaP cell as a control. Five probes for the 22Rv1 and seven probes for the LNCaP cells satisfied the described selection criteria.

### Mutations in the AS3 gene in 22Rv1 cells are likely to occur during the growth of the primary tumor

The androgen-induced AS3 gene, also known as regulator of cohesion maintenance, homolog B (S. cerevisiae) (PDSB5), has been shown to be a mediator of the androgen-induced proliferative arrest of human androgen sensitive prostate cells in culture and in the rat prostate *in situ *[14]. 22Rv1 cells containing two heterozygous inactivating frameshift mutations in the AS3 gene is a human prostate carcinoma epithelial cell line derived from a xenograft that was serially propagated in mice after castration-induced regression and relapse of the parental, androgen-dependent CWR22 xenograft [15]. We rationalized that mutations in the AS3 gene could be selected during propagation of CWR22 cells in castrated mice and thus might account for the tumor relapse in castrated mice, giving rise to the 22Rv1 variant. Sequencing of the CWR22 genomic DNA, however, has identified AS3 gene mutations identical to that in 22Rv1 (not shown), suggesting that mutations in the AS3 gene occurred during the growth of the original primary tumor and not during the propagation of CWR22 cells in castrated animals or the passaging of 22Rv1 cells in tissue culture. This result is in line with previously reported findings [7] demonstrating that each of the mutations detected in the 22Rv1 prostate cancer cell line was also detected in the CWR22 cells.

### Analysis of phenotype alterations produced by expression of the PARD3 gene in LNCaP cells

**Figure 2:**
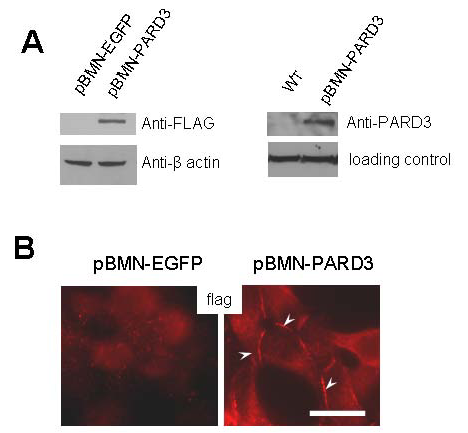
**Ectopic expression of Par3 protein in the LNCaP cells**. (A). Western blotting with anti-Flag (left) or with anti-PARD3 (right) antibody shows PARD3 expression in whole-cell lysates from the LNCaP cells stably transfected with Flag-tagged PARD3 expression vector but not in control nontransfected cells or transfected with EGFP empty vector. Blotting with anti-β-actin antibody was used as a loading control. (B) Immunofluorescent analysis with anti-flag antibodies shows localization of exogenous Flag-tagged Par3 protein at cell-cell contacts. Size bar is 20 μm.

The product of the PARD3 gene, which was first identified in *C. elegans*, is essential for asymmetric cell division and polarized growth. The protein has been reported to be involved in epithelial tight junction [16] and in cell-cell adherence junction [17]. To analyze the role of mutational inactivation the PARD3 gene for the LNCaP cells, we cloned the cDNA corresponding to the NM_019619.2 transcript variant of the RARD3 mRNA with the sequence of FLAG-tag attached to the 3' end into the pBMN-I-GFP retroviral expression vector. The FLAG-tagged PARD3 expression vector or empty vector (EGFP), was used for virus production and transfection of LNCaP cells. Western blot analysis with anti-FLAG or with anti-Par3 antibodies shows a protein band of predicted size in the LNCaP cells tranfected with the PARD3-Flag expression construct but not in control LNCaP cell not transfected with the construct or transfected with the empty EGFP vector (Figure 2A). Immunofluorescence analysis with anti-Flag antibodies has shown localization of the ectopically expressed Par3 protein along cell-cell contact regions (Figure 2B), in agreement with the reported involvement of Par3 protein in an epithelial tight junctions [16] and in an adherens junctions [18].

**Figure 3:**
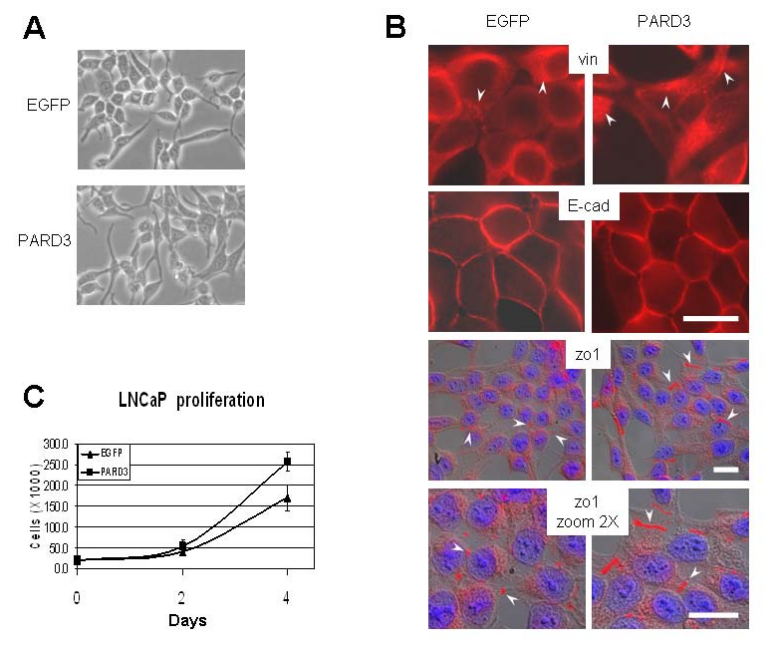
**Effect of Par3 protein expression on cell morphology, focal adhesion contacts, cell-cell contacts and proliferation rate**. (A). Phase contrast images (200×) of the LNCaP cells expressing or not expressing Par3 protein. (B). Immunoflurescent images showing the foci of vinculin (vin) - representative marker for focal adhesion contacts (stained with red indicated with arrows), E-cadherin - (E-cad) representative marker for adherens junctions (stained with red). Zonula occludens-1 (ZO-1) - representative marker for tight junctions is presented with overlay pictures from three independent layers: Differential Interference Contrast (DIC) visualizing cells shape, ZO-1 stained with red (indicated with arrows) and nuclei stained with blue. Size bar is 20 μm. (C). 5 × 10^3 ^cells per well were seeded in 24-well format tissue culture plate in RPMI 1640 containing 5% fetal calf serum (FBS). Cells were cultured for indicated time periods, harvested and counted by hemocytometer. Cell numbers are shown as the average ± S.D. of counting of cells in four wells per time-point. The experiment was repeated independently three times with similar results.

Examination with a light microscope did not detect significant differences in appearance between the LNCaP cells, ectopically expressing Par3 protein, and the LNCaP cells transfected with empty vector (Figure 3A). However, since slight alterations in cell shape still could be observed (Par3 expressing cells seem to look slightly more attached to the plastic) we investigated the results of ectopic Par3 expression on focal adhesion as well as on tight and on adherens junctions. Staining of control and Par3 expressing LNCaP cells with anti-vinculin antibodies has shown that focal adhesions was not affected by ectopic expression of Par3 (Figure 3B top). Neither had Par3 expression had an effect on adherens junction as shown by staining with anti E-cadherin antibody (Figure 3B middle). However, staining with anti-ZO-1 (used as a marker for tight junction) antibody has shown that tight junction might be affected by ectopic Par3 expression. Usually, the ZO-1 staining surrounds cells by continuous line when their tight junctions are well established. On the contrary, LNCaP cells are traced by ZO-1 staining with dots as a clear indication of not so well established tight junctions (Figure 3B EGFP bottom). The expression of Par3 increases partially the continuality of the ZO-1 signal which indicates some stabilization (but not restoring) of tight junctions (Figure 3B bottom).

**Figure 4:**
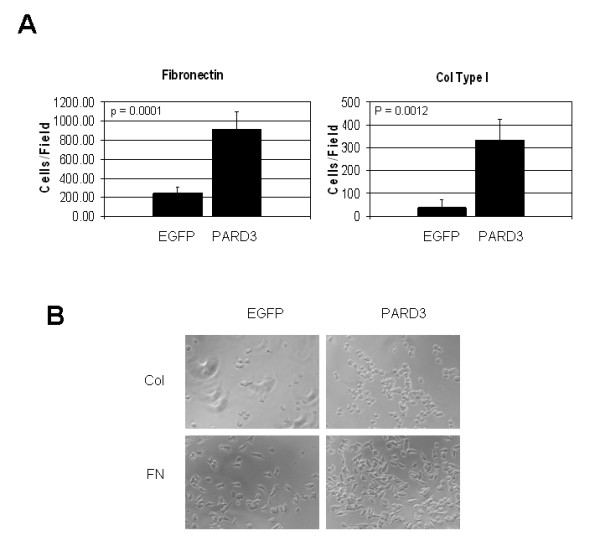
**Ectopic expression of Par3 protein enhances adhesiveness of the LNCaP cells**. (A). 2 × 10^4 ^of LNCaP cells stably expressing Par3 protein or transfected with empty vector (EGFP) were seeded over fibronectin or collagen Type I pre-coated wells of a 96-well plate and incubated at 37°C for three hours, as described in the "Methods" section. After washing the wells with Dulbecco's phosphate-buffered saline the numbers of attached cells were calculated as the average from eight wells. The results were presented as the mean value ± S.D. The experiment was repeated three times with similar results. (B). Representative phase-contrast pictures (200×) of the cells attached to wells coated with fibronectin (FN) and collagen Type I (Col) after performing adhesion assay as described above for panel (A).

We also found that expression of Par3 resulted in higher adhesion of LNCaP cells to the components of extracellular matrix collagen type I and fibronectin (Figure 4).

**Figure 5:**
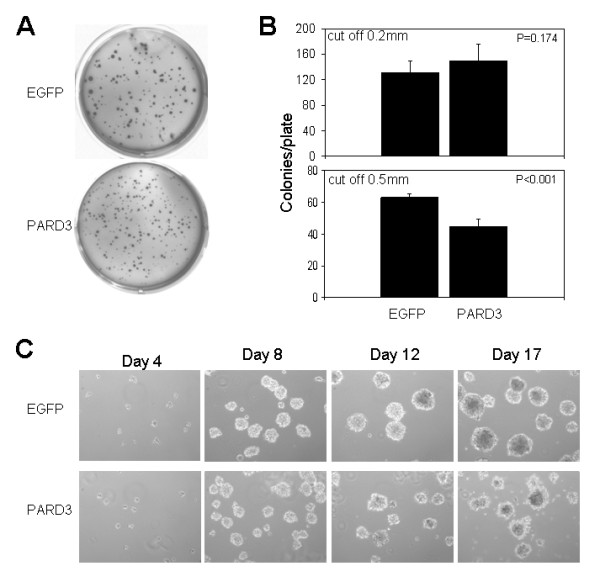
**Effect of expression of Par3 protein on anchorage-independent growth and on three-dimensional growth in Matrigel matrix**. (A). Representative images of the wells with colonies developed inside soft agar on day 21. (B). Charts represents the actual number of colonies per well with cut off diameter for counting - 0.2 mm and 0.5 mm. The experiment is representative of three independent analyses with similar results. (C). The effect of PARD3 expression on the three-dimensional growth of LNCaP cells in reconstituted basement membrane (Matrigel). Equal numbers of LNCaP cells expressing Par3 protein or control LNCaP cells transfected with empty vector (EGFP) were seeded over Matrigel pre-coated wells of 24-well plate plates in 2% Matrigel as described in "Methods". The phase contrast images (200×) were taken on every 4 days to track 3D structure formation. The images represent three independent experiments with similar results.

Contrary to our expectations, with regard to a possible tumor-suppressor role for the PARD3 gene, ectopic expression of the PARD3 gene in LNCaP cells resulted in a higher proliferation rate in tissue-culture (Figure 3C). On the other hand, anchorage independent growth in soft agar was inhibited by PARD3 expression. Figure 5A illustrates that although number of colonies with diameter >0.2 mm formed by PARD3-expressing cells was slightly higher than that formed by control LNCaP cells tranfected with empty vector, the number of larger colonies with diameter >0.5 mm was significantly lower in the PARD3-expressing cells (Figure 5B). Also we found that expression of PARD3 affected the growth of LNCaP cells in 3D culture in Matrigel. Figure 5C shows that colonies formed by LNCaP cells not expressing Par3 protein are larger and look more condensed, while the colonies formed by Par3 expressing cells look dispersed at the periphery.

## Discussion

We have previously demonstrated using GINI that LNCaP cells have biallelic inactivating mutations in the JAK1, CLPTM1 and SYNJ2 genes [13]. In this work, using an improved version of GINI, we have added the PARD3 to the list of mutant genes in the LNCaP cells. Our method has again identified the CLPTM1 as a candidate for sequencing but missed the JAK1 and SYNJ2 genes. The reason we missed the SYNJ2 may be that the cut- off threshold chosen for selecting candidates for sequencing were too stringent. Analyzing the microarray data in the Additional file 1: Table S1, one can see that loosing the stringency of selection parameters results in the appearance of the SYNJ2 gene in the list of candidates; however, the list is becoming too large. The JAK1 gene was missed because the 22Rv1 cells used as a control for the LNCaP also contained a mutation in the JAK1. Using an additional control cell line, such as PC3, for example, would result in including the JAK1 in the list of candidates, since PC3 has no mutation in this gene. The advantage of using many cell lines in GINI analysis has been demonstrated recently by identifying mutations in melanoma cell lines [19]. By analyzing a panel of 12 cell lines, the authors identified three novel melanoma tumor-suppressor genes. The melanoma cell lines analyzed do not have microsatellite instability and, consequently, must have low mutation frequency. This fact, besides suggesting the importance of mutant genes for the development of melanoma, demonstrates that GINI is not restrained to identifying mutations in MSI positive cell lines. Although applying our version of GINI to simultaneous analysis of multiple cell lines of the same tumor type could dramatically improve the efficiency of mutation identifications, false positives caused by cell line-specific variability in the efficiency of NMD degradation of transcripts representing the natural substrate of NMD put limit on GINI efficiency. Another limitation to GINI efficiency is the reported variability in the efficiency of NMD for the degradation of mutant mRNAs [20]. Finally, short-lived mRNAs transcripts degraded by other than NMD mechanisms will also be frequently overlooked in GINI analysis. With the advent of the next-generation sequencing, however, further improvement of GINI efficiency may become irrelevant.

The AS3 gene in the 22RV1 cells has been inactivated by frameshift mutations in the (C)7 and (A)9 mononucleotide repeats. Frameshift mutations at repetitive sequences in a coding DNA in cancers with MSI do not necessary indicate the tumor-suppressor role of the mutant genes, but may be just a byproduct of the inherent instability of repetitive DNA. However, the involvement of the AS3 gene product into androgen receptor signaling and vitamin D receptor action in prostate cancer cells [21] suggests that inactivation of AS3 may contribute to the development of androgen-independent prostate cancer.

The type of mutations in the PARD3 gene in LNCaP cells, a base substitution resulting in nonsense codon in one allele and the deletion of a (T) in a (T)5 repeat in the other allele, suggests the selective advantage provided by the mutational inactivation of the gene; a repeat as short as (T)5 is stable even in cancers with MSI [22,23] and two independent mutations in a gene is a highly improbable event to occur without selection. The higher proliferation rate of LNCaP cells following the ectopic expression of PARD3 gene indicates that the mutations were unlikely to occur during the propagation of LNCaP cells in tissue culture; the LNCaP cells without mutations in the PARD3 gene would quickly outgrow the cells with mutations in tissue culture.

Par3 protein is a member of the Par complex that includes Par3, Par6, serine/threonine kinase aPKC (atypical Protein Kinase C) and small GTPase Cdc42 [24-26]. The complex regulates epithelial cell-polarity and asymmetric cell division. Loss of cell polarity is thought to be involved in multiple aspects of oncogenesis, such as invasive and migratory processes [27,28], it is also suggested that deregulation of asymmetric cell divisions of stem or progenitor cells could be responsible for the abnormal self-renewal and differentiation of cancer stem cells [29,30]. Although restoration of PARD3 expression in LNCaP cells resulted in a higher rate of cell-proliferation in tissue culture, it also impaired the ability to form large, condensed colonies in a three-dimensional culture. This fact supports the idea that cell-polarity-mediated 3D tissue organization can function as a non-canonical tumor-suppressor [30]. Since the restoration of PARD3 expression in LNCaP cells did not suppress tumor growth in subcutaneously injected nude mice (data not shown) we can suggest that mutational inactivation of the PARD3 gene could be a tumor-initiating event and occur at the level of the prostate stem cell. PARD3 inactivation-mediated loss of asymmetric cell division could lead to accumulation of stem or progenitor cells and to disruption of 3D tissue organization, resulting in the initiation of tumor formation. Alternatively, PARD3 inactivation could be a late event in the LNCaP tumor progression. Since the expression of Par3 protein resulted in a higher adhesiveness of LNCaP cells to the components of the extracellular matrix, it is possible that the PARD3 inactivation, although reducing the proliferative potential at the same time promoted cell migration to the lymph node, from which the LNCaP cell line is derived.

## Conclusion

Using an improved version of GINI, we identified biallelic inactivating mutations in the AS3 and PARD3 genes in prostate cancer cell lines. Inactivation in a prostate cancer cell line of the PARD3 gene, involved in asymmetric cell division and maintenance of cell-polarity suggests that the loss of cell-polarity contributes to prostate carcinogenesis.

## Competing interests

The authors declare that they have no competing interests.

## Authors' contributions

II worked on the identification of mutant genes using GINI analysis. DK worked on the analysis of the biological effect of PARD3 expression in LNCaP cells, and YI coordinated the study and wrote the manuscript. All authors read and approved the final manuscript.

## Pre-publication history

The pre-publication history for this paper can be accessed here:

http://www.biomedcentral.com/1471-2407/9/318/prepub

## Supplementary Material

Additional file 1 **Table S1. Quintile normalized log transformed U133Plus2.0 GeneChip hybridization data on GINI analysis of LNCaP and 22Rv1 cells**. Affymetrix GeneChip Human Genome U133Plus2.0 arrays were used to analyze total RNA prepared from LNCaP and 22RV1 cells after the following treatments: 1) no treatment (the columns LNCaP Ctrl and 22Rv1 Ctrl); 2) four hours of incubation with caffeine (LNCaP Caff and 22Rv1 Caff); 3) four hours of incubation with caffeine followed by four hours of incubation with caffeine and Actinomycin D (LNCaP CAC and 22Rv1 CAC) and 4) four hours incubation with caffeine followed by four hours of incubation wit Actinomycin D (LNCaP CA and 22Rv1 CA). The Affymetrix .CEL files were loaded into an R software environment and quintile-normalized and log-transformed using the *gcrma *function, available at the http://www.bioconductor.org Click here for file
